# An Information-Theoretic Analysis of High-Frequency Load Disaggregation

**DOI:** 10.3390/e28030334

**Published:** 2026-03-17

**Authors:** Gabriel Arquelau Pimenta Rodrigues, André Luiz Marques Serrano, Geraldo Pereira Rocha Filho, Vinícius Pereira Gonçalves, Rodolfo Ipolito Meneguette

**Affiliations:** 1 Department of Electrical Engineering, University of Brasilia, Brasília 70910-900, Brazil; andrelms@unb.br (A.L.M.S.); geraldo.rocha@uesb.edu.br (G.P.R.F.); vpgvinicius@unb.br (V.P.G.); 2 Institute of Mathematical and Computer Sciences, University of São Paulo, São Carlos 13566-590, SP, Brazil; meneguette@icmc.usp.br; 3 Department of Exact and Technological Sciences, State University of Southwest Bahia, Vitória da Conquista 45083-900, BA, Brazil

**Keywords:** entropy, information theory, mutual information, NILM, random forest

## Abstract

High-frequency non-intrusive load monitoring provides detailed harmonic information for appliances’ power disaggregation, and machine-learning approaches have demonstrated good performance in this task. However, these methods provide little transparency regarding the information structure of the aggregate signal. To address this, this paper models NILM as a coding-decoding process and applies information-theoretic measures to quantify uncertainty, recoverability, temporal contribution, and inter-appliance masking effects in aggregate signals. In the analyzed dataset, transfer entropy suggests negligible temporal gains, which is consistent with the observed effectiveness of pointwise models such as Random Forest. Moreover, conditional mutual information emphasizes the asymmetric masking relationships between appliances, with the laptop charger acting as a dominant interferer in the considered measurements. These findings are validated through a Random Forest regression model with minimum Redundancy Maximum Relevance feature selection. The results show that the mutual information between an appliance and the aggregate is a good predictor of disaggregation performance in the examined data, as appliances with high mutual information, such as hair dryer and electric water heater, achieve lower estimation errors, while others, such as iron, are difficult to recover despite stable distributions. This relationship is statistically supported by a strong negative monotonic correlation between normalized mutual information and the disaggregation error (Spearman $r_{s}=-0.81$, p=0.015). Hence, this work demonstrates how information-theoretic analysis can help characterize disaggregation difficulty prior to model training and assess the observability of appliances in high-frequency NILM.

## 1. Introduction

Monitoring energy consumption separately for each appliance is relevant for smart grids, as it produces the data necessary to improve efficiency, reduce waste, and support sustainable electricity use [1]. However, this granular measurement is complex and expensive, as it requires dedicated sensors, which makes this approach impractical in environments with a large number of devices to monitor. To address this challenge, non-intrusive load monitoring (NILM) uses a single measurement point at the main electrical panel to infer individual appliance consumption. This technique, also known as load disaggregation, eliminates the need for sensors for each appliance while facilitating the analysis of the appliances’ consumption data, such as the detection of malfunctioning equipment and the management of peak demand.

High-frequency NILM uses detailed harmonic information from instantaneous voltage and current samples that are commonly lost in low-frequency smart meter data. This results in superior disaggregation accuracy compared to the low-frequency analysis, although the optimal sampling frequency may depend on the algorithm [2]. Indeed, NILM models have achieved high accuracy in load classification and disaggregation tasks, demonstrating that aggregate measurements can be decomposed into individual contributions [3,4].

Despite these advances, the research focus has mostly been on performance gains, with limited discussion about the information limits that determine appliance observability in aggregate measurements [5]. Consequently, practical evaluations of NILM in commercial environments have demonstrated significant performance degradation due to load multiplicity, continuous baseload, and ambiguous small power transitions [6].

These findings suggest that algorithmic improvements may be insufficient if the aggregate signal does not embed sufficient recoverable information. Information theory provides model-agnostic tools for analyzing these limits, as it quantifies uncertainty and statistical dependence between signals. Thus, a prior quantification of information content may help determine the observability limits before model deployment.

Therefore, this work interprets the NILM problem as a coding-decoding process, as individual appliance signals are encoded into an aggregate measurement through physical superposition, and disaggregation corresponds to the decoding of each appliance’s consumption from the measured signal.

This interpretation promotes an information-theoretic analysis of appliance observability [7]. This domain provides metrics such as Shannon entropy, that may quantify appliance uncertainty; mutual information, that measures the recoverable information from the aggregate; and the conditional mutual information, that quantifies masking effects between appliances. This analysis combines the fields of signal processing and information theory, thus providing observations that explain why certain loads achieve better disaggregation performance than others.

The dataset used in this work is the high-frequency NILM dataset proposed by [8], comprising eight appliances, namely, a hair dryer, an electric water heater, a hair straightener, a fridge, an iron, a screen, a laptop charger, and a lamp. The features corresponding to measurements of these appliances, along with those of the aggregate signal, are analyzed with the information-theoretic metrics and the findings are validated with a Random Forest NILM model.

The main objective of this study is to quantify how much information about the appliances is embedded in aggregate high-frequency measurements and to evaluate whether the metrics used can predict disaggregation performance prior to model training.

### 1.1. Contributions and Limitations

This work proposes an information-theoretic formulation of the NILM problem as a coding-decoding process, enabling a model-agnostic characterization of appliance observability from high-frequency aggregate measurements.

The study also demonstrates that information-theoretic observability metrics correlate with empirical disaggregation performance, showing that normalized mutual information predicts Random Forest regression error across appliances. This relationship advances the estimation of disaggregation difficulty and informs feature selection prior to model training. Additionally, the analysis shows that a small subset of odd-order harmonics is sufficient for high-frequency NILM, promoting efficient system design without significant performance degradation.

The proposed analysis is computationally lightweight and scalable, as the metrics are computed independently for each appliance. The computational cost depends mainly on the number of appliances and the number of observations in the dataset. Furthermore, since the approach does not require training complex models, it can be applied efficiently to larger datasets or households with a greater number of appliances.

Another advantage is that it does not require manual tuning of multiple parameters. The metrics are computed using discrete probability estimation, which reduces the need for empirical parameter adjustment and contributes to the robustness of the analysis.

As limitations, the analysis is conducted on a single dataset comprising eight appliances recorded in a controlled domestic environment, which restricts the generalizability of the findings to other appliance types, household configurations, and operating conditions. Also, the dataset’s approximately two-second sampling interval may hide temporal effects.

Additionally, the use of histogram-based probability estimation avoids the smoothing bias of kernel density methods, but introduces sensitivity to bin width selection.

### 1.2. Structure of This Work

The remainder of this article is structured as follows. Section 2 reviews similar and relevant works. Section 3 presents the proposed methodology of the work, whilst Section 4 discusses the information-theoretic results. Section 5 correlates the findings with a Random Forest model and Section 6 concludes this paper.

## 2. Related Work

Probabilistic approaches to NILM have been developed to disaggregate low-frequency smart meter data without relying on supervised training. In [9], an unsupervised Bayesian methodology is introduced to disaggregate household consumption into base components using prior distributions derived from seasonal patterns and environmental variables. The authors estimate load distributions with hourly resolution without requiring labeled appliance-level data. Classical probabilistic models such as hidden Markov models have also been adopted for unsupervised load disaggregation, modeling appliance state transitions as latent stochastic processes inferred from aggregate power signals [10]. A NILM model may also be based on deep learning architectures to learn mappings from aggregated signals to appliance-level consumption [11]. Hybrid architectures combining convolutional neural networks and long short-term memory models have also been proposed to represent spatial and temporal characteristics of appliance signals [12].

Load disaggregation has previously been considered as a coding-decoding problem, in which appliances are mapped to aggregate power values [13]. This approach introduces entropy of device states and mutual information (MI) of power values to quantify how distinguishable appliance combinations are. Similar to this perspective, our work proposes an information-theoretic analysis of disaggregation. However, we analyze high-frequency measurements including harmonic components and extend the analysis to characterize directional and conditional dependencies.

Elastic matching algorithms have also been proposed for energy load disaggregation, in which appliance signatures are matched to aggregated power frames without requiring parametric model training [14]. Five different techniques were evaluated on the REDD datasets, with minimum variance matching achieving the highest disaggregation performance. As a comparison, our work validates information-theoretic findings with disaggregation experiments using a Random Forest model, a machine learning-based approach.

In addition, other works have focused on supervised appliance classification, in which individual appliance signals are taken as input and classified into their respective appliance categories [15,16]. A two-stage event-based disaggregation framework has been proposed, in which appliance switching events are first detected using a $\chi^{2}$ goodness of fit test and subsequently paired to extract features from low-frequency active power measurements [17]. In the first stage, events are grouped based on phase information, steady-state power variation, and peak characteristics, while in the second stage a Support Vector Machine classifier is applied for appliance identification. Unlike these approaches, our work does not perform appliance classification, but estimates per-appliance power consumption directly from aggregated measurements. Neural-network approaches using appliance-specific architectures have also been proposed to improve identification accuracy [18].

Detailed appliance-level information inferred from aggregate measurements may also create privacy concerns, as disaggregation can reveal sensitive information such as occupancy patterns, daily routines, and behavioral habits of household members [19]. Consequently, several works have investigated privacy-preserving NILM techniques that aim to limit the exposure of appliance information while maintaining disaggregation performance [20,21]. These approaches demonstrate that the ability to recover appliance activity from aggregate signals is related to the potential for information leakage.

Although our work does not propose privacy-preserving mechanisms, the information-theoretic analysis is relevant to this context because it quantifies how much appliance-specific information is embedded in aggregate measurements. Hence, the proposed observability metrics may also inform the potential privacy exposure of smart meter data regardless of the disaggregation algorithm.

Table 1 compares this work with related literature. “High-frequency” indicates access to instantaneous voltage or current samples with sufficient resolution to reconstruct intra-cycle waveform characteristics. “Information-theoretic” refers to the use of concepts from Shannon information theory to support methodological decisions. “Energy disaggregation” involves estimating individual appliance power consumption from aggregated measurements, in which works limited to appliance identification or state classification are marked as partial.

## 3. Materials and Methods

**Figure 1:**
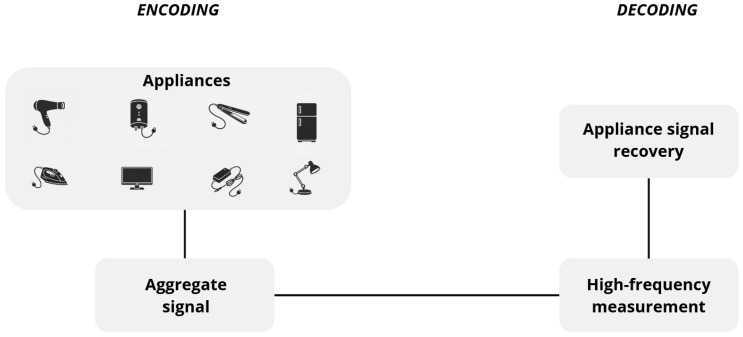
The proposed information theoretic approach to load disaggregation.

In this work, the NILM problem is formalized as a coding-decoding process, as illustrated in Figure 1. This section describes the methodology adopted to quantify the information content of the aggregate signal and assess its relationship with disaggregation performance. Python version 3.12.12 is used in the experiment.

Within this interpretation, the aggregate measurement may be formally modeled as a multi-source additive channel. Let $X_{i}\left(t\right)$ denote the power contribution of appliance *i* at time *t*, and let Y(t) represent the aggregate measurement. The encoding process is given by Equation (1), where ε(t) represents the unmodeled disturbances.

(1) $$Y\left(t\right)=\sum_{i=1}^{N}X_{i}\left(t\right)+\varepsilon \left(t\right),$$

In this formulation, each appliance $X_{i}$ acts as an information source and the aggregate signal *Y* corresponds to the channel output. The additive structure in Equation (1) can be interpreted as a multi-source communication process in which several independent generators simultaneously transmit their signals through a shared physical medium. The disaggregation algorithm acts as a decoder that attempts to recover each source signal from the observed channel output, and the degree to which this recovery is possible depends on the amount of information about each source that is preserved in the aggregate signal.

### 3.1. Dataset

The dataset used in this work, proposed by [8], contains measurements of root mean square current ($i_{\mathrm{rms}}$), root mean square voltage ($v_{\mathrm{rms}}$), power factor, apparent power ($p_{\mathrm{apparent}}$), active power ($p_{\mathrm{active}}$), and current harmonic amplitudes from the 1st to the 32nd order ($h_{1}$–$h_{32}$), sampled approximately every two seconds. The harmonic components are computed internally by the metering integrated circuit using a discrete Fourier transform engine operating at an 8 kHz sampling frequency over 0.5 s windows.

The dataset is separated into 16 recording sessions collected on different days and approximately 8 h long, each containing CSV files that correspond to a specific appliance. The monitored appliances are a hair dryer, an electric water heater, a hair straightener, a fridge, an iron, a screen, a laptop charger, and a lamp. The individual appliance measurements are recorded simultaneously with the aggregate circuit.

### 3.2. Information-Theoretic Metrics

To quantify appliance observability within the encoding-decoding interpretation of NILM, we adopt information-theoretic measures. Let $X_{i}$ denote the signal of appliance *i*, and *Y* the aggregate measurement.

The continuous power signals were discretized prior to probability estimation to enable the computation of Shannon entropy and related measures in discrete form. This is because the direct estimation of differential entropy from continuous data requires kernel or k-nearest-neighbor density estimators, which introduce smoothing parameters whose selection is non-trivial and may lead to unreliable estimates for signals with complex distributions.

To avoid this, histogram-based probability mass functions were constructed using the Freedman–Diaconis rule, which determines the bin width (*h*) according to Equation (2), where IQR denotes the inter-quartile range, and *n* the sample size. For appliance signals, the Freedman–Diaconis rule is applied exclusively to non-zero values to preserve the structure of appliance activity, with zero representing the OFF state.

(2) $$h=2\frac{\mathrm{IQR}}{n^{1/3}}$$

Although histogram-based estimators introduce sensitivity to bin width selection, the Freedman–Diaconis rule adapts the bin width to the dispersion and sample size of the data. In this work, the same binning strategy is applied consistently for all appliances and sessions to ensure comparability between the estimated probability distributions.

The variability of each appliance may be quantified in bits through Shannon entropy, defined in Equation (3). It represents the uncertainty associated with appliance activity, providing a reference scale for interpreting dependency measures.

(3) $$H\left(X_{i}\right)=-\sum_{x}p\left(x\right)\log_{2}p\left(x\right)$$

The statistical dependence between appliance $X_{i}$ and the aggregate signal *Y* is quantified by mutual information, given in Equation (4). This measures the reduction in uncertainty about $X_{i}$ given observation of *Y*. To enable comparison between appliances with different entropy levels, mutual information is normalized as expressing the fraction of appliance information recoverable from the aggregate signal, represented in Equation (5).

(4) $$I\left(X_{i};Y\right)=\sum_{x,y}p\left(x,y\right)\log_{2}\frac{p(x,y)}{p(x)p(y)}$$

(5) $$\mathrm{NMI}\left(X_{i};Y\right)=\frac{I(X_{i};Y)}{H(X_{i})}$$

The distributional variability across measurement sessions is quantified using the Jensen–Shannon distance ($d_{JS}$), which measures the difference between probability distributions associated with the same appliance under distinct recording conditions. The $d_{JS}$ is defined in Equation (6).

(6) $$d_{\mathrm{JS}}\left(P∥Q\right)=\sqrt{\frac{1}{2}D_{\mathrm{KL}}\left(P∥M\right)+\frac{1}{2}D_{\mathrm{KL}}\left(Q∥M\right)},\;M=\frac{1}{2}\left(P+Q\right)$$

In Equation (6), $D_{\mathrm{KL}}$ denotes the Kullback–Leibler divergence, defined in Equation (7).

(7) $$D_{\mathrm{KL}}\left(P∥Q\right)=\sum_{x}P\left(x\right)\log_{2}\frac{P(x)}{Q(x)}$$

The $d_{JS}$ provides a symmetric and bounded ($0\leq d_{\mathrm{JS}}\left(P∥Q\right)\leq 1$) measure of how distinct the statistical distributions of an appliance are in different sessions. Lower values indicate stable appliance signatures, whereas higher values reflect variability in the encoded signal.

To evaluate the temporal effect in each appliance, we compute transfer entropy (TE) from appliance signals to the aggregate measurement, according to Equation (8). This quantifies the additional predictive information provided by past appliance states in addition to the past of the aggregate itself. For considering short-term temporal dependencies, TE is evaluated for time lags τ∈{1,2,3,4,5}.

(8) $$T_{X_{i}\to Y}\left(\tau \right)=I\left(X_{i}^{t-\tau };Y^{t}|Y^{t-1}\right)$$

Ultimately, the appliance discernibility in the presence of other active loads is evaluated using conditional mutual information, which quantifies how much information about appliance $X_{i}$ remains in the aggregate signal when conditioning on another appliance $Z_{j}$, for i≠j. This metric is calculated as in Equation (9).

(9) $$I\left(X_{i};Y|Z_{j}\right)=H\left(X_{i}|Z_{j}\right)-H\left(X_{i}|Y,Z_{j}\right),$$

The conditional mutual information is then normalized by the unconditional mutual information $I(X_{i};Y)$. This ratio (ρ) expresses the fraction of information about appliance $X_{i}$ that remains in the aggregate after considering $Z_{j}$. A value of ρ=1 indicates that $Z_{j}$ does not mask $X_{i}$, while ρ=0 implies complete masking, with $X_{i}$ becoming indiscernible when $Z_{j}$ is active. Values exceeding unity (ρ>1) occur when conditioning on $Z_{j}$ reduces the uncertainty.

### 3.3. Load Disaggregation with Feature Selection

To investigate whether the information-theoretic observability metrics are predictive of disaggregation performance, a Random Forest regressor is trained per appliance and evaluated with varying feature set sizes. This model is adopted due to its previously high reported performance in this dataset [8].

Feature selection is conducted using the minimum Redundancy Maximum Relevance (mRMR) criterion, which ranks features by maximizing their mutual information with the target appliance signal and penalizing redundancy among selected features. Relevance is quantified as the mutual information between each aggregate feature and appliance power consumption, averaged across all appliances and sessions to obtain a balanced relevance measure. Redundancy is computed as the mutual information between pairs of aggregate features using pooled session data.

For a candidate feature *f*, the selection score is defined in Equation (10), where S denotes the set of already selected features.

(10) $$\mathrm{score}\left(f\right)=I\left(f;X_{i}\right)-\frac{1}{\left|S\right|}\sum_{s\in S}I\left(f;s\right)$$

We used mRMR to select subsets of size *k*, with *k* ranging from the total number of features down to one. Random Forest regression models were then evaluated for a subset of feature sizes k∈{1,2,…,20,25,30,35,37}.

For each evaluated feature subset size *k*, the regression models were trained using a Random Forest regressor with 100 trees. Then, a session-wise Group K-Fold cross-validation strategy is employed with K=5.

In this configuration, each recording session is treated as a group, and all samples belonging to the same session are assigned to the same fold. During each iteration, the model is trained using data from four folds and evaluated on the remaining fold. This procedure prevents samples from the same recording session from appearing simultaneously in the training and test sets, thus reducing cross-session leakage and providing a more realistic estimate of generalization performance across different recording conditions.

The performance of the model is evaluated using the coefficient of variation of the Root Mean Square Error (CVRMSE), computed as the ratio between RMSE and the mean of the appliance power values within the corresponding test fold, which scales the error relative to the magnitude of the target signal.

(11) $$\mathrm{CVRMSE}=\frac{\sqrt{\frac{1}{N}\sum_{t=1}^{N}{\left(P_{t}-{\hat{P}}_{t}\right)}^{2}}}{\bar{P}}$$

The CVRMSE is calculated by Equation (11), in which $P_{t}$ is the true power at time *t*, ${\hat{P}}_{t}$ is the estimated power, *N* is the total number of samples, and $\bar{P}$ is the mean true power over the evaluation period.

## 4. Information Structure of Appliance Signals

This section presents the results of the proposed information-theoretic metrics.

### 4.1. Static Information Content

The entropy of an appliance’s power consumption indicates its variability. A higher entropy is obtained from a more unpredictable consumption pattern and, consequently, a greater amount of information is needed to characterize its state. Conversely, a load with negligible entropy exhibits little variability and can be predicted with a trivial constant estimator, but its contribution to the aggregate carries no distinguishable signature, making it difficult to isolate from other loads.

Another consideration is the stability of entropy in different recording sessions. If an appliance exhibits consistent entropy over sessions, its statistical signature may be considered transferable in different operating conditions. Conversely, high inter-session variability in entropy suggests that the appliance’s behavior is irregular, which may complicate model generalization.

To measure this, we compute the entropy for all appliances in each session. Table 2 presents the inter-session entropy statistics computed for the appliances.

The results show that the laptop charger exhibits high entropy ($\mu_{H}=3.377$ bits) with low dispersion (${\mathrm{CV}}_{H}=0.151$ bits), indicating stable usage patterns with high information content. In contrast, appliances such as the iron and screen present lower mean entropy values, suggesting less variable activation behavior.

Moreover, the high coefficient of variation (CV) of appliances like the fridge and the lamp suggest a significant variability in their entropy among sessions. This dispersion indicates that, although average information content may appear moderate, individual sessions can deviate considerably.

This is evident from their minimum entropy values, which are zero for several appliances. For instance, in the session ‘05–20 8h’, the electric water heater, hair straightener, and iron remained constantly off, drawing no active power and thus resulting in zero entropy. Similarly, in the session ‘06–02 8h’, the fridge and lamp exhibited variations so small that the Freedman–Diaconis rule produced a single bin, hence zero Shannon entropy.

Entropy, however, does not quantify how much information the aggregate signal provides about each appliance, whose individual contributions must be inferred from it. To quantify this relationship, we use mutual information between the aggregate and each appliance for all sessions. The resulting values are normalized by the appliance’s entropy in the corresponding session, so that the mutual information represents the proportion of the appliance’s uncertainty that is reduced when the aggregate signal is observed. This normalization produces a dimensionless measure bounded between zero and one, where higher values indicate a stronger statistical dependence. Therefore, appliances with high normalized mutual information with the aggregate have a stronger statistical association with the measured signal. Within the analyzed dataset, this suggests that a larger fraction of the appliance variability is represented in the aggregate measurement, which may facilitate its disaggregation.

**Figure 2:**
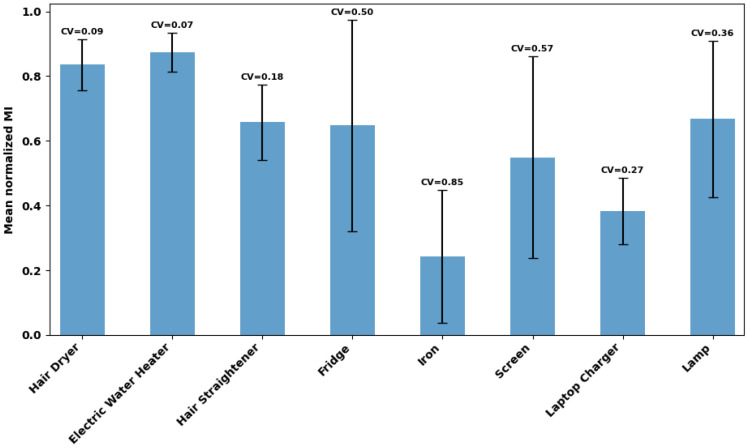
Mean normalized mutual information per appliance over sessions.

The mean normalized mutual information for each appliance is presented in Figure 2, disregarding sessions with null entropy to avoid division by zero. Because sessions with null entropy correspond to periods in which the appliance is inactive or with negligible variability, excluding them conditions the normalized mutual information statistics on sessions where the appliance presents observable activity. Consequently, the reported averages represent the information structure during active periods, and not across all recording sessions.

From these results, it can be inferred that hair dryer, electric water heater, fridge, and lamp exhibit higher mutual information, indicating that a larger fraction of their power variability is recoverable from the aggregate, which is expected to facilitate power estimation during active periods. Conversely, iron and laptop charger show lower values, indicating greater difficulty.

Furthermore, the hair dryer and electric water heater also have the lowest coefficients of variation, which confirms that their observability is stable in the recording conditions. In contrast, the highest CV of the iron indicates that its observability fluctuates over the sessions. The high dispersion for the fridge and screen suggests that their recoverability is dependent on the session, despite moderate mean normalized MI.

### 4.2. Variability Across Sessions

**Figure 3:**
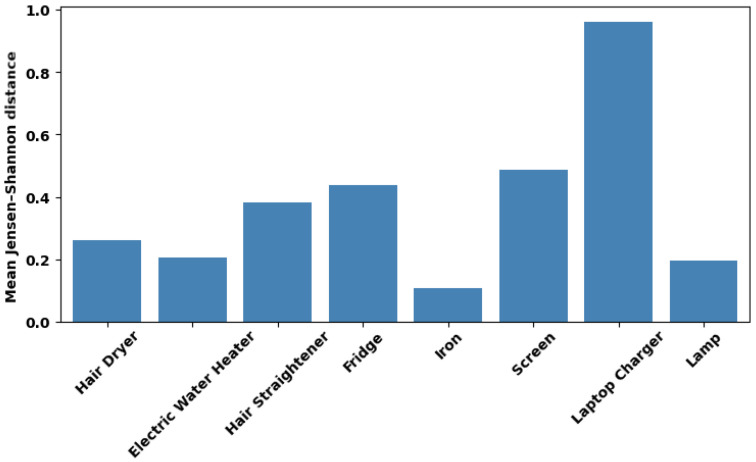
Mean $d_{JS}$ per appliance.

As seen in Section 4.1, the entropy and mutual information of some appliances are variable across sessions, indicating that their statistical properties are not stationary over time. To investigate this variability, we analyze the dissimilarity between the probability distributions of power consumption independently of the aggregate. For each appliance, we compute the Jensen–Shannon distance for all possible pairs of recording sessions, and the results are shown in Figure 3.

The laptop charger has the highest mean $d_{JS}$, approaching the theoretical maximum of 1.0, which indicates that its power distribution changes almost completely between sessions. One possible reason is that, since power draw is influenced by the battery level, the resulting distributions may be nearly non-overlapping among recordings. This is consistent with prior work showing that charger behavior changes significantly with battery level [26].

Conversely, the iron presents the lowest mean $d_{JS}$, which suggests that its power distribution is stable. It also has, however, a low mean normalized MI, as shown in Figure 2, which demonstrates that distributional stability of the appliance signal, despite being a desirable property for NILM, does not guarantee its recoverability from the aggregate.

**Figure 4:**
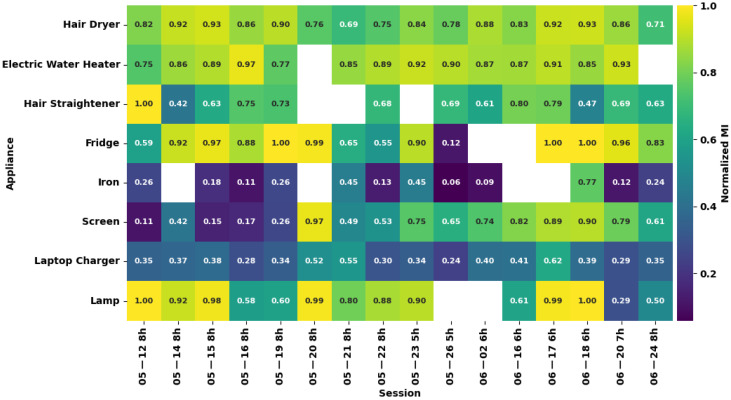
Heatmap of normalized MI between each appliance and the aggregate power consumption, computed per session.

Figure 4, which presents the normalized MI values per appliance per session, shows that the fridge’s dispersion is influenced by a single outlier session, whereas the hair dryer and electric water heater maintain consistent high normalized MI values. White squares in the figure correspond to sessions with null entropy.

Another observation is the temporal progression of the screen, which transitions from low normalized MI in early sessions to high values in later ones. This suggests that the screen’s relationship with the aggregate evolved over the course of data collection, possibly due to changes in co-occurring loads or usage intensity. For disaggregation model development, this implies that a model trained on early sessions would fail to generalize to later ones, and vice versa.

### 4.3. Temporal and Conditional Information

To quantify the influence of past appliance states on the current aggregate measurement, transfer entropy is used. This metric expresses the reduction in uncertainty about the current aggregate value when the past of an appliance is known, given the aggregate’s own history. Therefore, this analysis measures the additional information that temporal structure provides for appliance recovery, thus supporting the choice between pointwise and sequence-based disaggregation models.

**Figure 5:**
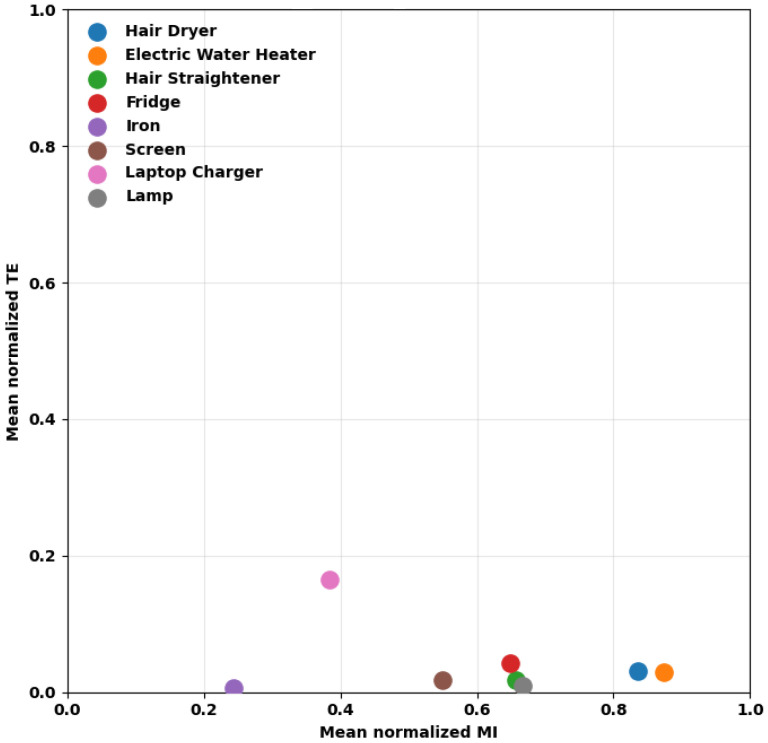
Transfer entropy per appliance for up to five lags.

The results shown in Figure 5 indicate that temporal dependencies are negligible for most appliances. This suggests that, for these loads, the knowledge of past appliance activity provides little additional predictive value. These findings align with those of [8], who concluded that Random Forest, a pointwise model using harmonic features, outperformed sequence-based architectures, such as a Convolutional Neural Network, for the same appliance types. Notwithstanding, it should be noted that the low transfer entropy observed for most appliances may be a consequence of the sampling resolution of the dataset.

Nonetheless, the temporal patterns and the dependence to the aggregate signal may be obscured when multiple appliances operate simultaneously. To address this, we examine conditional mutual information, which measures the remaining dependence between an appliance and the aggregate after considering the concurrent operation of other loads. This explains how appliances may mask one another in the aggregate measurement.

**Figure 6:**
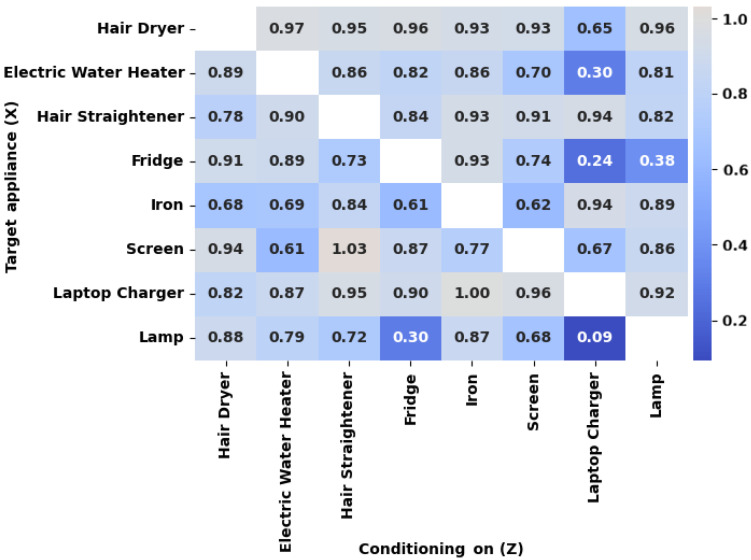
Conditional MI normalized by unconditional MI per appliance pair.

Figure 6 presents the information retention ratio $\rho =I\left(X_{i};Y|Z_{j}\right)/I\left(X_{i};Y\right)$ for all ordered appliance pairs, where rows correspond to the target appliance $X_{i}$ and columns to the conditioning appliance $Z_{j}$. Values close to 1.0 indicate that the target is discernible despite the concurrent operation of the conditioning appliance, whereas values near zero suggest that the target’s contribution to the aggregate is obscured when $Z_{j}$ is active.

It is noteworthy that the matrix is asymmetric, which confirms that the masking is directional and not mutual. For instance, conditioning the laptop charger on the presence of the lamp results in a near-zero retention ratio of 0.09, but the reverse pair retains 0.92 of its original information. This indicates that the lamp’s signature is almost completely suppressed by the laptop charger’s load, while the charger is discernible even when the lamp is active.

It is also observed that the laptop charger has low retention values with other appliances, indicating that, within the analyzed dataset, its operation compromises the observability of several other loads. This pattern suggests that certain appliances may reduce the discernibility of others when operating simultaneously.

These findings are confirmed by previous work, which noted that the concurrent operation of multiple loads causes overlapping signatures that prevent a NILM system from extracting appliance information from the aggregate, and that simultaneous events become indistinguishable as the number of active loads increases [6]. The masking relationships in Figure 6 provide a quantification of how concurrent loads suppress one another’s observability in the aggregate signal.

However, the temporal observations are measured considering the approximate 2 s sampling interval of the measurements, which may hide short temporal dependencies. Consequently, the limited temporal findings represent the characteristics of this dataset, and do not necessarily support a general conclusion about NILM systems.

## 5. Validation Through Disaggregation

The information-theoretic analysis of Section 4 characterizes the recoverability potential of each appliance and their interactions with the aggregate signal. To examine whether these metrics are consistent with empirical disaggregation behavior, we perform an additional analysis using a Random Forest regression model. This experiment is intended as an internal plausibility check that compares the information-theoretic observations with the performance of a practical NILM model.

With this purpose, we use a Random Forest regression model to perform energy disaggregation on the same dataset. The Random Forest is selected because it (i) achieves a good performance on this data; and (ii) is a simpler model that does not explore temporal dependencies, which, as discussed in Section 4.3, provide limited predictive information for most appliances. Nevertheless, it is noteworthy that the machine learning model is used only to validate the predictive relationship between the information-theoretic metrics and empirical disaggregation performance, and they are not used as input features for the model.

The Random Forest model is trained iteratively using feature subsets ranked by the mRMR method. It starts with the most important feature and additional features are progressively incorporated as per their importance order.

The features rank is presented in Table 3. It can be observed that the highest-ranked feature is the fundamental component ($h_{1}$) followed predominantly by odd-order harmonics. This pattern is corroborated by the literature on nonlinear electrical loads, that concludes that odd harmonics carry the most discriminative information for load disaggregation [27].

**Figure 7:**
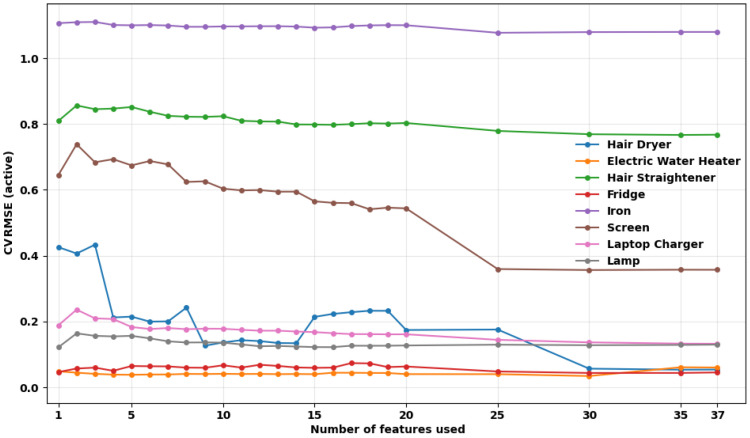
CVRMSE achieved by the Random Forest model for each appliance under different feature subset sizes, measured only during appliance active periods.

Figure 7 shows the CVRMSE as a function of the number of features incrementally added according to the mRMR ranking. It is noted that some appliances experience substantial error reduction within the first 10 features, whilst others benefit from the inclusion of higher ranked variables.

The hair dryer and the electric water heater, for example, achieve low CVRMSE values even with few features. This reinforces the findings from Section 4.1 that their observability is high and from Section 4.2 that they are stable across sessions.

The iron also has high CVRMSE values, with little improvement as features are added. This behavior corroborates its low normalized MI, indicating weak recoverability from the aggregate.

An additional observation is that the disaggregation mostly improves as features are added, indicating that the Random Forest is robust to the inclusion of lower-ranked variables. However, the lack of significant improvement beyond the top-ranked harmonics suggests that high-frequency NILM systems may operate efficiently with a small harmonic subset, reducing computational cost.

To evaluate the computational benefits of feature reduction, we measured training time and peak memory usage of the Random Forest models for different subset sizes. The measurements were obtained during the same Group K-Fold evaluation procedure and averaged across all appliances and folds. All experiments were conducted in a machine equipped with an Intel Xeon CPU @ 2.20 GHz (2 vCPUs) and approximately 13 GB of RAM.

The results, presented in Table 4, indicate that models using small harmonic subsets can significantly reduce computational requirements and maintain comparable disaggregation accuracy, supporting the observation that performance saturates after the inclusion of the most informative harmonic components.

**Figure 8:**
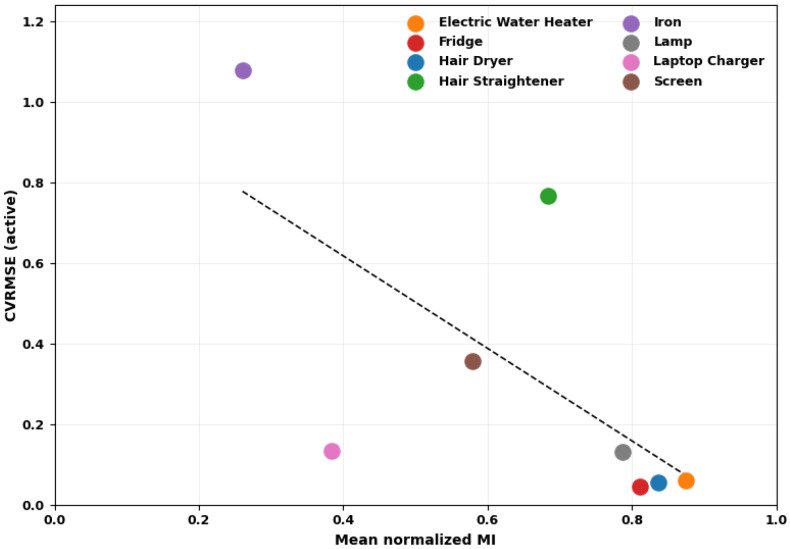
Correlation between mean normalized mutual information and active-period CVRMSE. The dashed line represents the linear regression line fitted to the data points.

To confirm that the proposed metrics are predictive of disaggregation performance, we analyze the relationship between the mean normalized mutual information and the active-period CVRMSE obtained with the full feature set (k=37), with the correlation being presented in Figure 8. The CVRMSE values correspond to the average performance across the Group K-Fold cross-validation procedure (K=5), computed over appliance active periods only.

A strong negative monotonic association is observed between normalized mutual information and disaggregation error, with Spearman’s rank correlation coefficient $r_{s}=-0.810$ (p=0.015<0.05). This statistically significant result indicates that appliances whose consumption patterns are more strongly represented in the aggregate signal achieve lower estimation error.

## 6. Conclusions and Future Works

This paper presented an information-theoretic approach for analyzing high-frequency load disaggregation, considering the NILM problem as a coding-decoding process. The static information analysis shows that the appliances of the used dataset differ in the recoverability of their signatures. The hair dryer and electric water heater have high normalized mutual information with the aggregate across sessions, indicating stable and recoverable signatures, which is observed in the high performance of the disaggregation of these appliances. Conversely, the iron presented low recoverability and a high disaggregation error.

The temporal analysis via transfer entropy shows that, within this dataset, past appliance states contribute negligible additional predictive information for most loads beyond what the aggregate’s own history already provides. This result offers a theoretical justification for the empirical observation that pointwise models, such as Random Forest, outperform sequence-based architectures on this type of data. The conditional mutual information analysis also indicates that the laptop charger acts as a dominant interferer that suppresses the observability of co-occurring loads.

These results suggest that information-theoretic metrics may elucidate disaggregation difficulty prior to model training, which promotes an informed algorithm design. Another implication is that high-frequency NILM systems may operate using few harmonics, as performance gains beyond the top-ranked features are small, and that past observations offer limited additional predictive information.

Therefore, the proposed analysis supports the design of NILM systems prior to model deployment and helps identify which loads are likely to be observable, as it quantifies the amount of recoverable information about individual appliances in aggregate measurements. This assists the evaluation of the feasibility of load disaggregation and informs decisions about sensing resolution or measurement configurations before investing computational effort in training complex models.

Future work could extend this analysis to other datasets and scenarios with different appliances, investigate its applicability at other sampling frequencies, and explore how the identified masking relationships can be explicitly exploited in the architecture of disaggregation models. Additionally, future study could adopt differential entropy to model appliance signals directly in the continuous domain.

## Figures and Tables

**Table 1:** Comparison with related works. Symbols denote: ✗ No, ∘ Partial, ✓ Yes.

Reference	Year	High-Frequency	Information-Theoretic	Energy Disaggregation
[13]	2015	✗	✓	✗
[14]	2020	✗	✗	✓
[16]	2020	✓	✗	∘
[22]	2021	✓	✗	✓
[23]	2021	✗	✓	∘
[24]	2023	✗	✓	✗
[25]	2025	✓	✓	∘
Our work	2026	✓	✓	✓

**Table 2:** Inter-session entropy statistics per appliance.

Appliance	$\mu_{H}$ (bits)	$\sigma_{H}$ (bits)	${\mathrm{CV}}_{H}$	Median (bits)	Min (bits)	Max (bits)	${\mathrm{CI}}_{95\%}^{\mathrm{low}}$ (bits)	${\mathrm{CI}}_{95\%}^{\mathrm{high}}$ (bits)
Hair Dryer	0.631	0.376	0.596	0.535	0.183	1.683	0.430	0.831
Electric Water Heater	0.439	0.271	0.616	0.366	0.000	0.864	0.295	0.583
Hair Straightener	0.570	0.476	0.835	0.567	0.000	1.577	0.316	0.824
Fridge	0.585	0.999	1.707	0.008	0.000	2.581	0.053	1.117
Iron	0.139	0.130	0.935	0.145	0.000	0.525	0.070	0.208
Screen	0.221	0.240	1.084	0.157	0.003	0.971	0.093	0.349
Laptop Charger	3.377	0.510	0.151	3.243	2.449	4.363	3.105	3.649
Lamp	0.296	0.400	1.353	0.114	0.000	1.134	0.083	0.509

**Table 3:** Feature ranking according to the mRMR method.

Rank	Feat.	Rank	Feat.	Rank	Feat.	Rank	Feat.
1	$h_{1}$	11	$h_{7}$	21	$h_{3}$	31	$h_{4}$
2	$h_{29}$	12	$h_{17}$	22	$h_{22}$	32	$h_{16}$
3	$h_{23}$	13	$h_{28}$	23	$h_{18}$	33	$h_{20}$
4	$v_{rms}$	14	$h_{27}$	24	$h_{12}$	34	$h_{10}$
5	$h_{13}$	15	$h_{11}$	25	$h_{5}$	35	$p_{active}$
6	$h_{31}$	16	$h_{6}$	26	$h_{2}$	36	$i_{rms}$
7	$h_{19}$	17	$h_{9}$	27	$h_{14}$	37	$p_{apparent}$
8	$h_{25}$	18	$h_{32}$	28	$h_{30}$		
9	$h_{26}$	19	$h_{15}$	29	$h_{8}$		
10	$h_{21}$	20	$h_{24}$	30	power_factor		

**Table 4:** Computational resource consumption for different feature subset sizes.

Number of Features Used	Mean Training Time (s)	Peak Training Memory (MB)
1	7.65	15.32
5	69.89	12.59
10	150.33	15.29
20	325.04	20.69
30	532.94	26.09
37	546.34	63.52

## Data Availability

This work uses a publicly available dataset, available at https://github.com/fariddinar/nilm-dataset [8] (accessed on 14 March 2026).

## Abbreviations

The following abbreviations are used in this manuscript:

CV	Coefficient of Variation
CVRMSE	Coefficient of Variation of the Root Mean Square Error
MI	Mutual Information
mRMR	Minimum Redundancy Maximum Relevance
NILM	Non-Intrusive Load Monitoring
TE	Transfer Entropy
